# Predicting strength distributions of MEMS structures using flaw size and spatial density

**DOI:** 10.1038/s41378-019-0093-y

**Published:** 2019-11-04

**Authors:** Robert F. Cook, Frank W. DelRio, Brad L. Boyce

**Affiliations:** 1 Materials Measurement Science Division, Material Measurement Laboratory, National Institute of Standards and Technology, Gaithersburg, MD 20899 USA; 2 Applied Chemicals and Materials Division, Material Measurement Laboratory, National Institute of Standards and Technology, Boulder, CO 80305 USA; 3 Materials Science and Engineering Center, Sandia National Laboratories, Albuquerque, NM 87185 USA

**Keywords:** Structural properties, NEMS

## Abstract

The populations of flaws in individual layers of microelectromechanical systems (MEMS) structures are determined and verified using a combination of specialized specimen geometry, recent probabilistic analysis, and topographic mapping. Strength distributions of notched and tensile bar specimens are analyzed assuming a single flaw population set by fabrication and common to both specimen geometries. Both the average spatial density of flaws and the flaw size distribution are determined and used to generate quantitative visualizations of specimens. Scanning probe-based topographic measurements are used to verify the flaw spacings determined from strength tests and support the idea that grain boundary grooves on sidewalls control MEMS failure. The findings here suggest that strength controlling features in MEMS devices increase in separation, i.e., become less spatially dense, and decrease in size, i.e., become less potent flaws, as processing proceeds up through the layer stack. The method demonstrated for flaw population determination is directly applicable to strength prediction for MEMS reliability and design.

## A framework for finding flaws

An analytical strategy for determining the size and distribution of structural flaws could enable the design of more robust microelectromechanical systems (MEMS)-based devices. The durability and design flexibility of these constructs is profoundly affected by both the size and spatial distribution of defects that form during the fabrication process. Researchers led by Robert Cook of the US National Institute of Standards and Technology have profiled the flaws in a series of polycrystalline silicon-based MEMS constructs. Through a combination of strength testing and atomic force microscopy analysis, the researchers were able to obtain valuable information about the distribution and structural impact of flaws that arise during MEMS component production. These insights could lead to more accurate predictions of the performance and durability of MEMS devices in future manufacturing efforts.

## Introduction

Three predominant factors motivate determination of strength-limiting flaw populations in manufactured materials. First, if flaws are defects that limit strengths of manufactured components, determination of a flaw population can be used to optimize manufacturing *yield*. Second, if flaws develop over time to become defects that limit lifetimes of operational components, determination of a flaw population can be used to predict *reliability*. Third, if flaws limit the size or shape of innovative components, determination of a flaw population can be used to extend *design* parameters. The second and third points are particularly important for engineered microsystems. Specifically, in the case of microelectromechanical systems (MEMS), the number of commercially manufactured MEMS placed in operation is very large (many billions) relative to the much smaller number (thousands at most) of MEMS-scale components that can be sampled and tested economically to determine a flaw population^1^. Hence, the required information leverages are extremely large for MEMS reliability prediction and design: There are clear performance consequences if a MEMS component is under-designed or reliability is over-estimated. Conversely, there are also clear commercial consequences if components are over-designed or reliability is under-estimated, or if yield is deliberately diminished by “proof testing”^2^.

A common method of assessing flaws, as well as providing strength information directly, is to measure the strengths of specially fabricated specimens sampled from a manufactured material. The sampled strength information is then used to infer flaw population features, especially flaw size. In this regard MEMS provide advantages and disadvantages. An advantage is that the micromachining techniques used in MEMS manufacturing are easily and economically able to fabricate statistically viable numbers of strength test specimens. A disadvantage is that the small dimensions of MEMS can lead to specimen handling issues in strength and other mechanical testing. Innovative designs have largely overcome this disadvantage and enabled experimental measurements of elastic modulus^3,4^, fracture toughness^5^, fatigue lifetime^6^, and, especially, strength^7–17^. In many experiments, large numbers of MEMS-scale components have been measured with great precision, and an extensive review of MEMS strengths, including assessments of modulus and toughness, highlights these advances^18^. In addition, recent works have developed and demonstrated clear analytical linkages between the probability distributions describing such experimentally determined strengths and the underlying manufactured populations of flaw sizes^19,20^. However, flaw populations are specified by *two*, essentially independent, attributes—size *and* spacing. The probability distributions developed in the recent works provided clear indications of large spacing, small spatial density, of strength-controlling flaws in MEMS and ceramics^19,20^. Earlier work^13^ utilized atomic force microscopy (AFM) of MEMS component surface topography to infer probable flaw type and spacing. However, no clear procedure has been developed to determine the spatial density of strength-controlling flaws in MEMS components.

The work here will demonstrate determination of size and spatial density attributes of material flaw populations in multiple polycrystalline silicon (polySi) layers formed in a MEMS manufacturing process. Nanoengineering or micromachining of MEMS is a layer-by-layer process and knowledge of layer-specific flaw populations is thus required for yield, reliability, and design optimization. Specially designed and fabricated groups of MEMS-scale strength test specimens of different geometries will be used. Clear distinction is made between the large flaw population in a total volume of manufactured MEMS material and the smaller numbers of flaws in groups of test specimens sampled from this population. Material flaw populations will be characterized by two attributes: (1) the size distribution of flaws, given as a probability density function (pdf), *f*, as a function of flaw size. Here crack length, *c*, will be used as the flaw size, such that the fundamental pdf characterizing the population is *f*(*c*); and, (2) the spatial arrangement of flaws, given here as the average separation Δ*L* between flaws along the length of a MEMS component (*e.g*., along a sidewall^11,13^). The reciprocal (1/Δ*L*) is thus the average lineal flaw density (flaws/length) in a MEMS component. It is the goal of this work to specify *f*(*c*) and Δ*L* for three different polySi layers from a MEMS manufacturing process, thereby enabling design and reliability predictions for arbitrary MEMS structures. The fundamental principles relating flaw populations and observed strength distributions have been described extensively in the recent analytical works^19,20^, including comparisons with commonly used descriptions. The current work is an *application* of these principles.

## Materials and methods

### Tensile and notched specimen fabrication

**Fig. 1:**
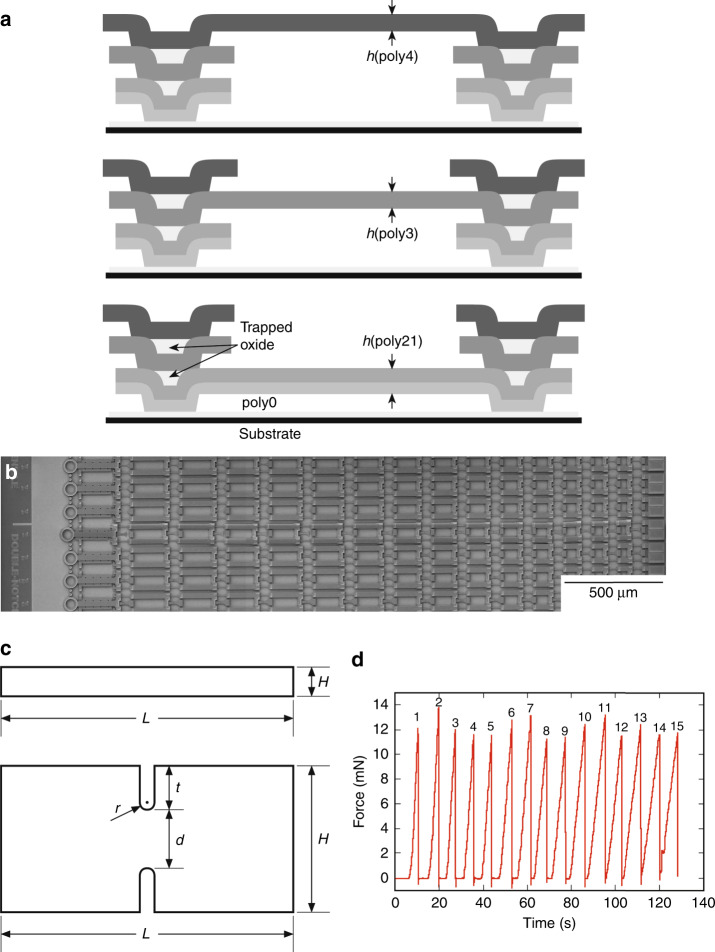
Tensile and notched specimen structure and behavior. **a** Schematic cross sections of the three polycrystalline Si MEMS layers used to create specimens for strength testing. In all figures, tensile loading is horizontal along the long axis of the specimens. **b** SEM plan image of several slack chain MEMS test structures used for strength testing. The ring pull tab for loading is visible at left. **c** Schematic plan views of the tensile and notched specimens with dimensional parameters indicated. **d** Representative force-time failure sequence for a tensile chain; individual specimen failures are numbered

The materials examined were polySi layers microfabricated using the SUMMiT V^TM^ process as in earlier studies^11,21^. In this process, five layers of polySi: poly0, poly1, poly2, poly3, and poly4, and four interlayers of oxide are deposited and lithographically patterned into MEMS structures. The layers exhibit near-identical microstructures of predominantly columnar grains with cross sections in the range 0.1 μm to 1 μm extending through a layer thickness and displaying no preferred crystallographic orientation^21^. In the earlier studies, involving a few tens of specimens per layer, a clear trend in strength, poly4 > poly3 > poly2 > poly1 was observed^11,21^, partially motivating the current work. Here, three materials—a composite poly21 layer, the poly3 layer, and the poly4 layer—were each formed into two different strength test specimen geometries—“tensile” bars and “notched” bars—using the SUMMiT V^TM^ reticle set RS733 in a single fabrication run. In addition, full-thickness three-layer specimens optimized for AFM measurements of sidewall topography were formed. The thickness, *h*, of each layer was about 2.3 μm; exact values determined from parametric monitoring during fabrication are given in Table 1. Figure 1a shows schematic cross sections of the layers. The tensile and notched test specimens were fabricated on single Si wafers as chains of specimens with single terminating pull tab rings^12^, shown in the scanning electron microscope (SEM) plan image of multiple chains of notched specimens, Fig. 1b. Some RS733 poly3 tensile bars were tested earlier^13^. The nominal overall width × length, *H* × *L*, of the specimens was 2 μm × 20 μm and 10 μm × 20 μm for the tensile and notched configurations, respectively. The notched specimens included straight-walled double edge notches with semicircular roots, radius *r*, about 0.5 μm and notch depths *t*, about 2.7 μm, to leave remaining section widths *d*, about 4.3 μm, slightly less than half the specimen width, *d* = *H* − 2*t*. Labeled specimen dimensions in schematic plan view are shown in Fig. 1c.

**Table 1:** MEMS polysilicon specimens and dimensions

Material, Specimen	Layer thickness, *h* (μm)	Specimen width, *H* (μm)	Notch depth, *t* (μm) Section width, *d* (μm)	Notch radius, *r* (μm)	Stress concentration factor, *K*_t_	Number of specimens, *N*
poly21, tensile	2.39	1.92 ± 0.02 (52)	–	–	–	483
poly3, tensile	2.33	1.74 ± 0.04 (77)	–	–	–	531
poly4, tensile	2.35	1.41 ± 0.06 (79)	–	–	–	576
poly21, notched	2.39	9.90	2.73 (4.44)	0.55	2.57 ± 0.05	331
poly3, notched	2.33	9.82	2.75 (4.33)	0.60	2.45 ± 0.07	284
poly4, notched	2.35	9.67	2.85 (3.98)	0.69	2.24 ± 0.08	435

**Fig. 2:**
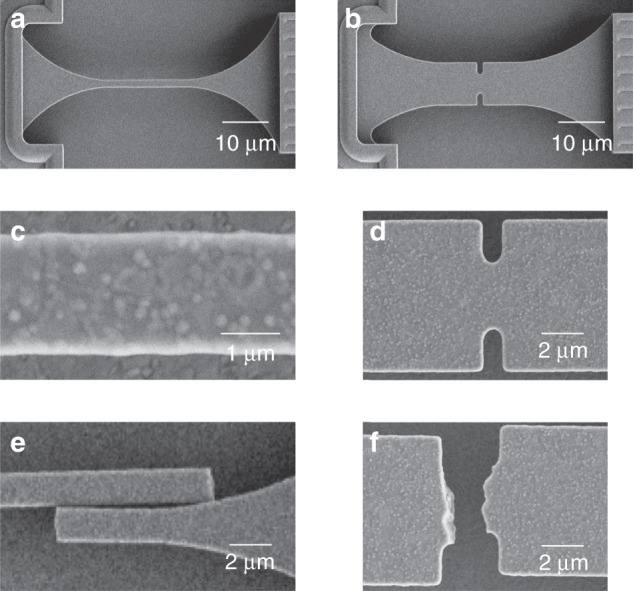
Detail images of tensile and notched specimens before and after failure. **a** SEM plan image of a poly3 material tensile bar as part of a slack chain testing structure. The side elements visible either side of the bar provide support after the bar is broken. **b** SEM plan image of a poly3 material notched bar as part of a slack chain testing structure. **c** Enlarged SEM plan image of a poly3 material tensile bar. Polycrystalline microstructural features and sidewall roughness are visible. **d** Enlarged SEM plan image of a poly3 material notched bar. **e** SEM plan image of broken poly3 material tensile bar illustrating brittle fracture. **f** SEM plan image of broken poly3 material notched bar illustrating notch failure

Figure 2a, b shows SEM plan images of single tensile and notched specimens formed in poly3; those formed in poly21 and poly4 were similar. Figure 2c, d shows enlarged SEM plan images of the tensile and notched specimens. The microstructure of the poly3 material is evident in both images and manifest in the small sidewall undulations or roughness visible in the image of the tensile specimen. The roughness is a consequence of etching during thermal and chemical steps in the manufacturing process and gives rise to dimensional variation or dispersion in all specimens. To characterize this dimensional dispersion, five SEM-based width measurements were made on 50–70 tensile specimens for each layer. Table 1 gives the mean ± standard deviation, *H* ± Δ*H*, of the measured width distributions and the number of measured specimens. The mean values *H* are all somewhat less than the target widths of 2 μm; Δ*H* was only a few percent of the mean widths, testifying to the reproducibility of the manufacturing process. Representative dimensions of the notched specimens are also given in Table 1, including the overall and section widths, *H* and *d*, and notch radii and depths, *r* and *t*. A key feature of the work here is that all specimens were fabricated identically such that only macroscopic shape (tensile or notch) varied between specimens formed at each layer. Microscopic surface roughness arising during fabrication thus remained invariant. Hence, the dimensional dispersion values determined from the extensive tensile specimen width measurements also pertained to the notched specimens. Examination of Table 1 shows that the dispersion of the notched specimen dimensions is near negligible with the exception of notch radius. The dimensional dispersions associated with roughness are not readily visible in the images of the notched specimens in Fig. 2d.

### Strength testing

Strength testing was performed by extending the pull tab ring, Fig. 1b, for each chain and recording the sequential peak failure forces, *F*.^12^ In each sequential failure, the force–displacement behavior was linear, indicating brittle failure by the propagation of a single crack, as shown in the representative poly4 tensile specimen chain force-time trace of Fig. 1d. Examples of such failed tensile and notched specimens are shown in the SEM images of Fig. 2e, f, respectively. Tensile specimen failure occurred randomly in the tensile gauge. Notched specimen failure occurred solely within the notch. Failure stresses, or strengths, *σ*, for the tensile specimens were determined by *σ* = *F*/(*Hh*), recognizing that (*Hh*) is the mean cross-sectional area of a tensile specimen. The uncertainties in the mean tensile specimen strengths were given by Δ*σ* = *σ*(Δ*H*/*H*), recognizing that the uncertainties in *F* and *h* were negligible and that mean strength uncertainty reflects dimensional dispersion. Mean failure stresses for the notched specimens were calculated from *σ* = *K*_t_*F*/(*dh*), where (*dh*) is the minimum section area at the notches, *F*/(*dh*) is thus the section stress, and *K*_t_ (>1) is the stress concentration factor (SCF) characterizing the enhanced stress at a notch root. The representative notched specimen dimensions in Table 1 were used to calculate the mean values of *K*_t_ = *K*_t_(*t*/*H*, *t*/*r*) from tabulated equations and treating the polySi layers as isotropic continua^22^. The dispersions in the SCF values, Δ*K*_t_, were determined by using the same analysis to calculate the maximum effects of dispersions in the notched specimen dimensions. The uncertainties in the mean notched specimen strengths were given by Δ*σ* = *σ*(Δ*K*_t_/*K*_t_), noting that this is a conservative upper bound to uncertainty as it assumes no correlation in notched specimen dimensions; as expected, dispersion in *r* had the greatest effect on notched strength uncertainty. Table 1 gives *K*_t_ ± Δ*K*_t_ for the notched specimens for each layer. The dispersions Δ*K*_t_ were a few percent of the mean values.

The number of specimens, *N*, in each of the six groups of strength tests (three materials × two geometries) are also given in Table 1; ~400 specimens/group. An empirical distribution function (edf) using *N* to describe each group of strength tests was formed^19,20^: The mean strengths for all specimens within each group were ranked, *σ*_1_, *σ*_2_, *σ*_3_, …, *σ*_*i*_, …, *σ*_*N*_, where *σ*_1_ was the smallest strength, *σ*_*N*_ was the largest, and *i*, 1 ≤ *i* ≤ *N*, was the rank. Associated with each strength was the rank parameter *P*_*i*_  = (*i* − 0.5)/*N*, 0 <  *P*_*i*_ < 1, such that a specimen randomly selected from the group has probability *P*_*i*_ of exhibiting a strength less than *σ*_*i*_. The discrete function *P*_*i*_ (*σ*_*i*_) was the group-specific edf. The edf provides a discrete estimation of the continuous cumulative distribution function (cdf) of strengths sampled from the large material population.

### Strength distribution and flaw population analysis

The strength edf for each group was fit by a continuous sigmoidal function, expressed parametrically as

1a $$F\left( \mu \right) = 30\left[ {(\mu ^{3p}/3) - (\mu ^{4p}/2) + (\mu ^{5p}/5)} \right],$$

where

1b $$\mu = (\sigma - \sigma _{{\mathrm{th}}})/(\sigma _u - \sigma _{{\mathrm{th}}}),$$

providing a bounded smoothing function for numerical analysis^20^. The strength parameters *σ*_u_ and *σ*_th_ provide upper and lower bounds, respectively, to the domain of the function, and the parameter 0 ≤ *μ* ≤ 1 gives the relative position within the domain. The parameter *p* is an empirical fitting parameter of order unity that controls the sigmoid symmetry. Although the shape of the sigmoid within the domain depends on *p*, key properties of *F(μ*) are *F*(0) = 0 and *F*(1) = 1 (set by the fixed prefactor 30) and *Fʹ*(0) = *Fʹ*(1) = 0 and these do not alter. Eq. 1a, b were best-fit to the edf for each group as described below. The derivation from the incomplete beta function and the above advantages of Eq. 1a, b in terms of boundedness and separation of shape from the properties of the bounds are discussed in detail elsewhere^20^.

*F*(*μ*) functions for the groups of notched specimens were applied in two ways: (1) to estimate the flaw-size population pdf *f*(*c*) and (2) to estimate the flaw spacing Δ*L*. These separate pieces of information were then brought together to simulate the physical tensile specimens in a manufactured material for comparison with independent observations. The cdf for a flaw population, *F*(*c*), is related to the cdf from a group of strengths, here described using *F*(*μ*), by^19,20^

2 $$F\left( c \right) = [1 - F\left( \mu \right)]^{{\mathrm{\Delta }}L/L},$$

where Δ*L* is the spatial separation of flaws in the population and *L* is the length of the specimens in the group. Length was used here as the specimen size characteristic, consistent with the extensive observations of sidewall failure origins in these materials^11–13^. Application of Eq. 2 to estimate the flaw population requires a relationship between crack length *c* and strength *σ* (normalized as *μ*), here taken as the Griffith equation^23^

3 $$\sigma = Bc^{ - 1/2} \cdot$$

A domain of crack lengths *c*_min_ ≤ *c* ≤ *c*_max_ gives rise to a conjugate reversed domain of strengths σ_*u*_ ≥ σ ≥ σ_th_, reflecting the inverse relationship of Eq. 3. The minimum threshold strength *σ*_th_ corresponds to the maximum crack length *c*_max_ and *vice versa*. Application of Eq. 2 also requires the ratio Δ*L*/*L*, here taken as 1/2, consistent with observation that stress concentrating effects ensured failure from a notch, independent of the size of the flaw; the effective length of the double edge notched specimens was thus *L* = 2Δ*L*. Once *F*(*c*) was determined from Eq. 2, *f*(*c*) was obtained by

4 $$f\left( c \right) = dF(c)/{\mathrm{d}}c$$

and the uncertainty in *f*(*c*) estimated from the notched strength uncertainty.

Extension of Eq. 2 to two groups of tensile specimens of different lengths, *L*_1_ and *L*_2_, sampled from the same flaw population but exhibiting different strength distributions, *F*_1_(*μ*) and *F*_2_(*μ*), gives

2b $$F\left( c \right) = [1 - F_1\left( \mu \right)]^{{\mathrm{\Delta }}L/L_1},$$

2c $$F\left( c \right) = [1 - F_2\left( \mu \right)]^{{\mathrm{\Delta }}L/L_2} \cdot$$

Eliminating Δ*L* from Eq. 2b, c gives,

5 $$F_2\left( \mu \right) = 1 - [1 - F_1\left( \mu \right)]^{(L_2/L_1)},$$

enabling distribution *F*_1_(*μ*) obtained from specimens of length *L*_1_ to describe distribution *F*_2_(*μ*) for specimens of length *L*_2_ through the ratio *L*_2_/*L*_1_. If specimen 2 is larger than specimen 1, i.e., *L*_2_ > *L*_1_, then distribution *F*_2_ will be narrower than distribution *F*_1_ as shown earlier^19^ and intended here: Recognizing from above that for notched specimens *L*_1_ = 2Δ*L* and for tensile specimens *L*_2_ = 40 μm, the flaw spacing is given by Δ*L* = (20*L*_1_/*L*_2_) μm. Eqs. 1 and 5 were fit to the tensile specimen strength data using the notched specimen strength data as a basis and *L*_2_/*L*_1_ as a fitting parameter. Δ*L* for each layer material was then determined using the above relation. The uncertainty in Δ*L* was estimated from the tensile strength uncertainty.

The quantitative characteristics of the three material layers, *f*(*c*) and Δ*L*, were used to simulate the physical surfaces by perturbing the rectangular outlines of the tensile specimens shown in Fig. 1c by the flaw populations. Plane geometry was assumed such that the specimen side walls were represented by the two tensile specimen edges. Flaw locations along the *L* = 20 μm edges, spaced Δ*L* apart, were identified. Crack lengths, *c*, were randomly selected from the population pdf *f*(*c*) and assigned these locations and surface openings of 0.1*c*. Visualizations of the cross-sections of the sidewalls and edges were then generated by perturbing the rectangular outline using the information in Tables 1 and 2 and representing the cracks as straight-sided. The cracks are barely visible at the scale of the diagrams in Fig. 1c.

**Table 2:** MEMS polysilicon layers strength distribution and flaw population parameters

Material Layer	Lower bound strength, *σ*_th_ (GPa)	Upper bound strength, *σ*_u_ (GPa)	Strength distribution exponent, *p*	Length scaling factor, *L*_2_*/L*_1_	Flaw spacing, Δ*L* (μm)	Grain boundary spacing, λ (μm)
poly21	1.95	4.35	1.9	70	0.29 ± 0.06	0.32 ± 0.02
poly3	2.00	4.20	1.8	35	0.57 ± 0.24	0.55 ± 0.06
poly4	2.60	5.35	1.9	13	1.53 ± 1.06	1.21 ± 0.23

### Surface topography measurement and analysis

AFM sidewall topography maps were used to provide confirmation of the Δ*L* values estimated from the strength measurements. Height maps, 2 μm × 4 μm, 256 pixels × 512 pixels, of the full-thickness three-layer specimens were obtained using intermittent contact mode AFM with a Si tip of radius 7 nm, similar to, but more extensive, than earlier work^13,19^. Approximately 20 maps extending over entire tensile specimen lengths were generated for each layer. The raw data peak-to-valley height range for all three layers was (75 ± 15) nm and the rms roughness values were (13.1 ± 1.9) nm, (9.8 ± 1.9) nm, and (8.2 ± 1.4) nm, for poly21, poly3, and poly4, respectively (uncertainties represent standard deviations from the multiple map measurements). The local curvatures of the topographic heights in each map were calculated by first smoothing the height data in one dimension along the image rows using a 3-pixel wide moving average followed by determination of the principal curvatures in two dimensions at each point using a 3 pixel × 3 pixel array. Maps of the maximum curvatures, 1/*ρ*, were generated, similar in appearance to those reported earlier^13^, but much more clearly revealing the sidewall grain structure as continuous lines of significant curvature. The linear intercept method was then applied visually on the curvature maps to determine the average separation, λ, along the length of the specimen between lines of significant maximum curvature. The independent spatial separation measurements, λ and Δ*L*, were then compared for each layer.

## Results

**Fig. 3:**
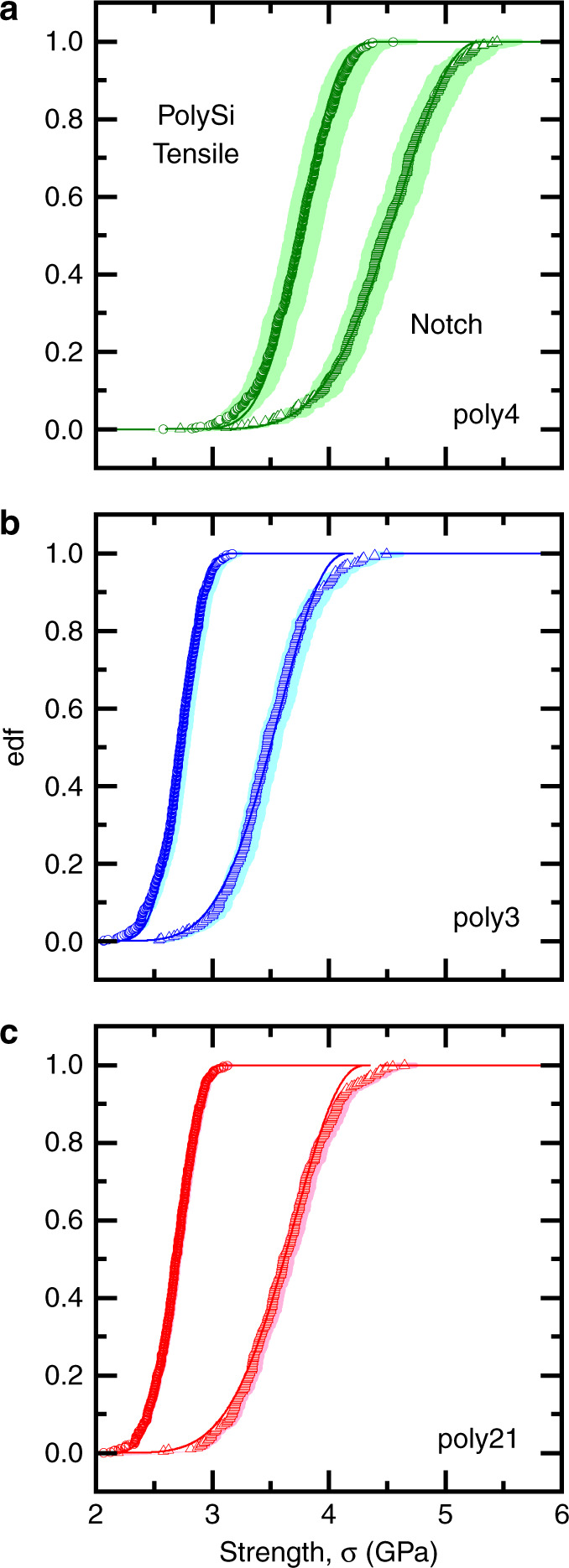
Empirical distribution function (edf) plots of failure stress for the three layers of MEMS materials shown in Fig. 1. Symbols and shaded bands represent means and uncertainties from experimental measurements from tensile and notched specimens shown in Fig. 2. Solid lines are best-fits of smoothing function

Figure 3 shows the experimental strength measurements of the notched and tensile specimens as edf plots. The symbols represent strength values for individual specimens calculated from the mean dimensions and SCF values given in Table 1. The sigmoidal edf curves and strengths of several GPa are typical for MEMS specimens^11,16,19^. For each layer the notched specimens were stronger than the tensile specimens, although the lower strength tails for each group were nearly coincident, leading to narrower tensile strength distributions than notch strength distributions, as anticipated by Eq. 5. Poly4 was stronger than poly3 and poly21, which were similar. The shaded bands in Fig. 3 represent uncertainties in the mean experimental values derived from the dimensional and SCF uncertainties given in Table 1. The notch strengths have greater uncertainties than the tensile strengths, reflecting the greater dispersion of the notch radii relative to the tensile widths. The uncertainties decrease in the order poly4, poly3, poly21, reflecting the overall decrease in relative dimensional dispersion. The upper solid lines for each set of strengths in Fig. 3 represent best fits of *F*(*μ*), Eq. 1, to the notch strengths subject to the constraint that the threshold strengths, *σ*_th_, were less than the smallest observed *tensile* strengths for each layer. The upper bounds, *σ*_u_, and shape parameters, *p*, were not constrained.

**Fig. 4:**
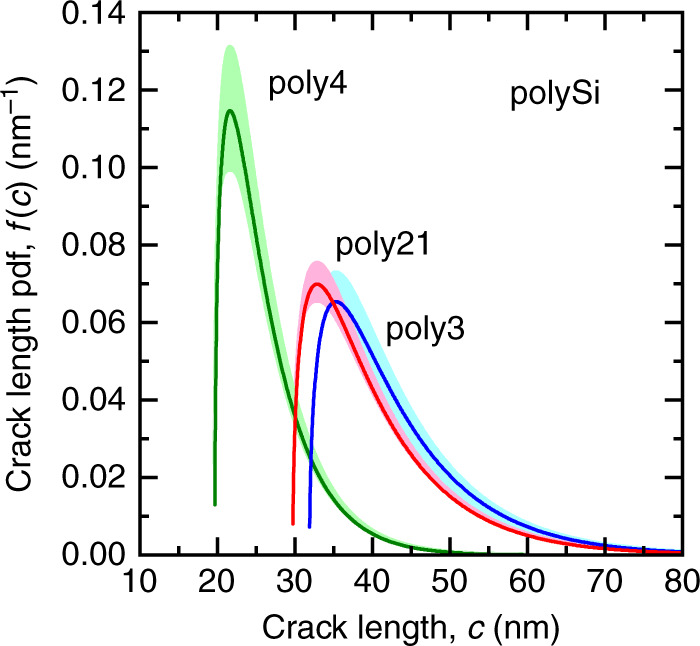
Probability density function (pdf) plots of flaw populations derived from the strength measurements of Fig. 3 for the three layers of MEMS materials shown in Fig. 1. Solid lines represent mean responses, shaded bands represent uncertainties

Using the toughness-related parameter *B* = 0.75 MPa m^1/2^ appropriate to Si^18^ and Eqs. 2 to 4, the flaw population pdf, *f*(*c*), was determined from *F*(*μ*) for each layer and is shown in Fig. 4. The solid lines in Fig. 4 reflect the mean responses and the shaded bands reflect uncertainties in the crack lengths arising from the uncertainties in strengths shown in Fig. 3. In all three cases, the pdf is asymmetric, consisting of many small flaws and an extended large flaw tail. The flaws are in ranges of 20 nm or 30 nm to ~80 nm, comparable to the dimensional dispersions. The *f*(*c*) plots for poly21 and poly3 are similar, reflecting the similar strengths, Fig. 3, and at smaller values for poly4, reflecting the overall larger strengths, Fig. 3.

Using the length ratio *L*_2_*/L*_1_ as a fitting parameter, Eq. 5 was used to fit tensile strength edf curves using the notch edf *F*(*μ*) parameters. The mean best fits to the tensile data are shown as the lower solid lines in Fig. 3. In each case, Eqs. 1 and 5 describe the tensile strength variations well within experimental uncertainty over the strength ranges, confirming a single flaw population. Table 2 gives the mean fit parameters *L*_2_*/L*_1_ for the tensile strengths; the values decrease monotonically through the layers from poly21 to poly4. A consequence of this decrease is that the mean flaw spacing increases through the layers from poly21 to poly4. The uncertainty in the flaw spacing was obtained by fitting non-linear Eq. 5 to the bounds of the strength uncertainties in Fig. 3 to gain 0.24 μm < Δ*L*(poly21) < 0.35 μm, 0.38 μm < Δ*L*(poly3) < 0.87 μm, and 0.69 μm < Δ*L*(poly4) < 2.86 μm. Table 1 gives the mean flaw spacing and approximations of uncertainty based on these bounds. Note that these inferred flaw spacings are comparable to the notch radii given in Table 1, providing strong support for the assumption above that the notches isolate a single flaw. The agreement between the bounds and the radii thus illustrates self-consistency between material flaw population deconvolution within a single series of strength tests, leading to Fig. 4, and strength scaling with specimen geometry between series of strength tests, Fig. 3.

**Fig. 5:**
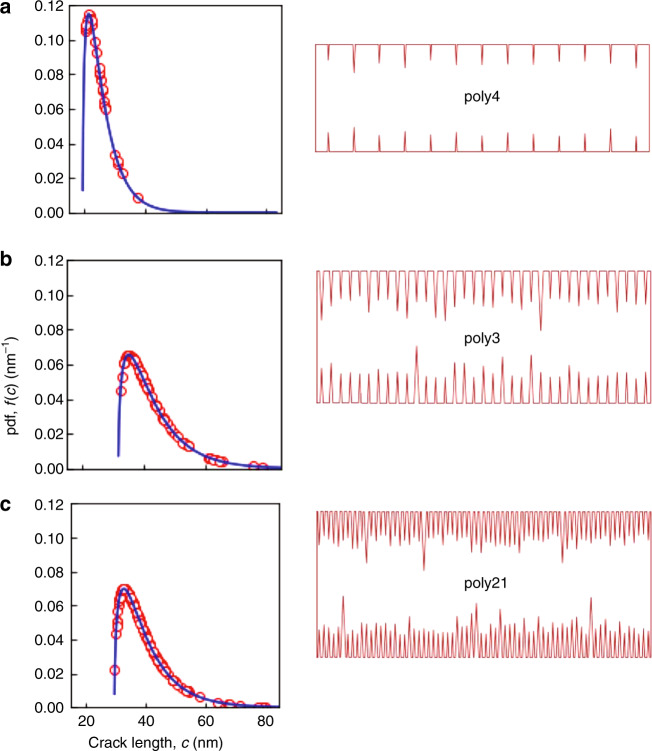
Probability density function (pdf) plots of flaw populations for the three layers of MEMS materials. Solid lines are mean responses from Fig. 4; symbols represent cracks randomly selected from the populations. Simulated schematic plan diagrams representing tensile specimens of the three layers using the selected cracks and spacing information deduced from strength tests. **a** poly4, **b** poly3, **c** poly21. Cracks magnified by a factor of 100 for visualization

The best fit mean *f*(*c*) responses and Δ*L* values were used to simulate the tensile specimen surfaces. The graphs in Fig. 5 show as solid lines the mean *f*(*c*) curves describing the flaw population in each layer, repeating the plots from Fig. 4. The symbols in Fig. 5 superposed on these lines represent flaws randomly selected from the populations at the crack lengths indicated, predominantly near the short crack peak of the pdf rather the long crack tail. Fewer flaws were selected in the order poly21, poly3, poly4 reflecting the decreasing *L*_2_*/L*_1_ ratios from Table 2. The schematic diagrams associated with each plot in Fig. 5 show the conjugate rectangular outlines and surface flaws of the simulated tensile specimens. For visualization purposes, the width/length ratios from Table 1 have been exaggerated by a factor of 10. The slight narrowing of poly4 relative to poly21 is visible. Perturbing the outlines in Fig. 5 are the selected cracks spaced Δ*L* apart. For visualization purposes the crack length/specimen length ratio has been exaggerated by a factor of 100. Many repeat instances of the simulations generated plots and images similar to the examples shown in Fig. 5.

Figure 5 makes clear the physical similarities and differences of the three material layers and the effects on strength. Inspection of Fig. 5b, c shows that poly21 and poly3 have about the same distributions of flaw sizes but have different flaw populations due to different flaw densities. In fact, poly3 has about half the average flaw density of poly21. In a 20 μm long tensile specimen, however, the difference in the mean flaw density becomes insignificant given the similar flaw size distributions, implying that the largest flaw in the specimen is likely to be similar and thus the strengths of poly21 and poly3 specimens are likely to be similar. This is the case in Fig. 5, in which the poly3 specimen has half the flaws of the poly21 specimen but the largest flaw in both specimens is about 80 nm. By contrast, Fig. 5 shows that the poly4 flaw population differs in *both* the distribution of flaw sizes and average flaw density. For example, poly4 has about five times the flaw spacing of poly21 and the flaws exhibit a markedly different size distribution with many smaller flaws. In this case, in a 20 μm tensile specimen, the difference between the mean flaw spacing is significant given the different flaw distributions implying that the largest flaw in the specimen is likely to be much smaller and thus the strength of poly21 and poly4 specimens is likely to be different. This is the case, as shown in Fig. 5, in which the poly4 specimen has not only many fewer flaws than the poly21 specimen but the largest flaw in both specimens is different, about 40 nm in poly4. It is reiterated that the distributions and images in Figs. 4 and 5 are not in any way schematic but are results from analysis of Fig. 3.

**Fig. 6:**
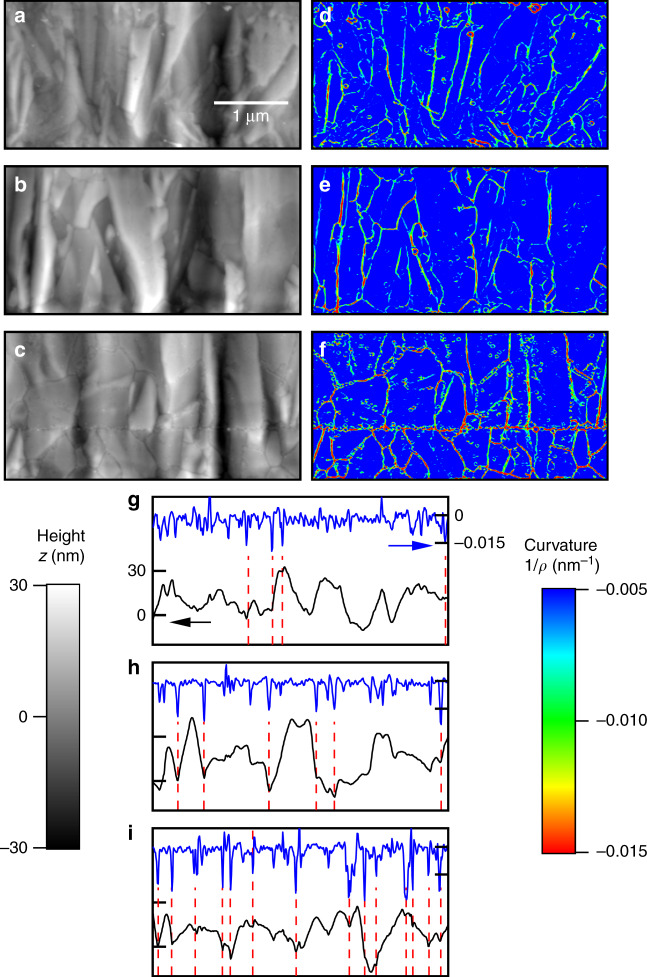
Surface maps and scans of the three MEMS layers. Topographic maps, **a** poly4, **b** poly3, **c** poly21, maximum curvature maps **d** poly4, **e** poly3, **f** poly21 and curvature line scans, **g** poly4, **h** poly3, **i** poly21, for the three layers of MEMS materials. Dashed lines on linescans indicate the intercepts used to estimate flaw spacing

Independent confirmation of the quantitative results of Figs. 4 and 5 is shown in the AFM measurements of Fig. 6. Figure 6a, b, c shows representative sidewall topography maps, height *z*, of poly4, poly3, and poly21, respectively, illustrating the similarity in overall appearance, peak to valley range, and rms roughness of the three layers. In more detail, Fig. 6d, e, f shows maps of maximum curvature, 1/*ρ*, of the same regions. Values of significant curvature form lines and patterns that very closely resemble the columnar grains and grain boundaries typical of MEMS polySi microstructures^21,24,25^ and follow the topography maps (the horizontal feature in Fig. 6c, f is the poly1-poly2 interface). It is clear that the curvature maps delineate grain-boundary surface grooves and that the grooves become more widely spaced and less distinct in the order poly21, poly3, poly4. The grooves are predominantly perpendicular to the layer surfaces and the applied stress in tensile tests (both horizontal, Figs. 1 and 2) and are thus associated with strength-limiting flaws^25^. The average separation of these grooves in the tensile direction thus provides an independent measure of flaw separation. Figure 6g, h, i shows topographic height and resultant curvature values taken from horizontal line scans 400 nm from the bottom of the maps, illustrating the above phenomena. Dashed lines indicate the large values of curvature, here taken as |1/*ρ*| > 0.015 nm^−1^, associated with grain boundary grooves and flaws that form the basis for analysis. Approximately 140 linescans were examined for each layer and the average separations of grooves, λ, estimated from linear intercept analysis and given in Table 2. The uncertainty in λ is the standard deviation of the measurement for each layer and thus provides a measure of dispersion in λ. It is clear that the mean values of λ from direct topographic measurements correlate extremely well with those of Δ*L* from analysis of strength measurements, well within the experimental dispersions, and also with the columnar grain sizes inferred from the earlier SEM cross-sections^21^. The correlations strongly support the idea that highly curved sidewall grain boundary grooves are the locations of strength-limiting flaws in this MEMS material. The implication is that the major intent of this work was fulfilled—the spatial density of flaws determined by strength testing was demonstrated and verified by the AFM measurements.

## Discussion

The methodology developed here for interpreting strength distribution measurements of micro-scale components is a powerful tool that extends the usefulness of strength testing in many ways. In particular, the methodology enables quantitative determination of the two key attributes of a flaw population—the distribution of flaw sizes and the average flaw density. The demonstration here specified the distribution of flaw sizes by the flaw population pdf, *f*(*c*), and the average flaw density by the flaw spacing, Δ*L*. For both attributes, measures of uncertainty were provided related to the uncertainty in the underlying strength measurements and component dimensional dispersions. The combination of both attributes enabled simulation of the physical surfaces of tensile strength specimens including the flaw size and spacing. The clear physical basis and analytical development of the methodology enables both internal consistency checks and tests, for example comparison of measured strengths with predicted strengths, and comparison and verification with independent measurements, for example comparison of measured topography and predicted surface topography. The methodology enables a more mechanistically-meaningful ability to extrapolate strengths to different stressed volumes and MEMS designs than the flaw size histograms developed earlier^8,11,13^ or the flaw sizes and spacings inferred from indentation tests^26^ and scanning probe measurements^11,13^.

In materials engineering application, the demonstration of the methodology here has provided an assessment of the refinement of flaw sizes and densities during the sequential formation of polySi layers during MEMS fabrication. The differences in the flaw populations of poly21, poly3, poly4 (at least for this SUMMiT V^TM^ process) are made especially clear in the diagrams of Fig. 5. Qualitatively, poly21 has a very dense array of large flaws, poly3 has a less dense array of large flaws, and poly4 has a sparse array of small flaws. This information can be used by a MEMS manufacturer to optimize heat treatment and etch processes to manipulate sidewall roughness and grooving during fabrication, beyond the strength observations noted earlier^11^. The observations here suggest that less thermal and chemical exposure leads to fewer, less potent sidewall flaws associated with the columnar layer structure. Quantitatively, the flaws in poly21 are most commonly about 35 nm in size and ~0.3 μm apart, those in poly3 are also about 35 nm in size but ~0.6 μm apart, whereas those in poly4 are most commonly about 20 nm in size and ~1.5 μm apart. This information can be used in quantitative MEMS reliability predictions^15^ and MEMS designs, especially when linked to size distribution information, Fig. 4, and strengths, Fig. 3. Similar information could also be used in the less common, less potent case of strength-controlling top-surface flaws (e.g., Fig. 2), discussed elsewhere^18^.

In a materials science sense, the phenomenon demonstrated here of notched specimens exhibiting strength distributions greater than those of tensile specimens is an aspect of “size effects”, usually considered for mechanical behavior of ductile materials—here extended to brittle materials. The stress concentrating effects of notches reduced the volume of stressed material in the mechanical tests such that the entire flaw population was assessed, including the smallest flaws exhibiting the largest strengths. In the absence of notches, the increased volume of tensile specimens led to stochastic sampling of the largest flaws in the population and a consequent contraction of the strength distribution towards the smallest strengths. Similar phenomena are observed in ductile metals: The stress concentrating effect of a small indenter reduced the volume of stressed material beneath spherical contacts on Mo such that the entire dislocation population was assessed, including those with the least mobility exhibiting the largest indentation “pop-in” loads and inferred shear stress^27–29^. As the indenter radius was increased, the increased volume of stressed material beneath the indenter also increased, leading to stochastic sampling of the more mobile dislocations in the population and consequent contraction of the pop-in distribution towards the smallest shear stresses. Indentation stress field volume was used to scale the pop-in stress for one set of large ductile strengths^27^ in much the same way that MEMS component length was used here for large brittle strengths. Similarly, the yield strengths of Cu pillars were observed to exhibit a broad distribution when the pillars contained a grain boundary such that the entire population of dislocations was assessed, including those with slip restricted by the boundary^30^. For single crystals with no boundary, the unrestricted increased volume led to stochastic sampling of the more mobile dislocations and consequent contraction of the yield stress distribution towards the smallest stresses.

In materials science application, the methodology is flexible enough to address the issue of the nature of the strength-controlling flaws. The flaws here were considered simple Griffith cracks and the strength-flaw size relation Eq. 3 was easily implemented. Examination of Fig. 6 provides support for the estimated scale of the flaws, the peak magnitudes of the groove radii, *ρ*, are 30–70 nm, comparable to the effective crack lengths, *c*, in Fig. 4. However there appears to be a negative correlation between *c* and *ρ* as the stronger poly 4 exhibits smaller *c* and larger *ρ* (smaller curvature, 1/*ρ*) suggesting that the nature of the flaws is probably somewhat different from the assumed simple Griffith cracks. The observations are consistent with both (i) small cracks at the roots of rounded grooves, as suggested previously^15^, in which case a fracture mechanics modification to Eq. 3 is required, and (ii) small, rounded grooves with no discernible crack “tip,” as also suggested^11,13^. In this latter case, Eq. 3 can simply be replaced by a different power-law relation for strength that is less sensitive to groove size than crack length but that requires connection to theoretical strength and elastic anisotropy^18^ rather than material toughness^31^. A combination of the materials science and engineering aspects is to model flaws as grooves generated during processing and then apply the resulting groove population to predict strength distributions of various sized components^32^. This is “forward” analysis as opposed to the reverse analysis methodology^19^ used here.

An important aspect of the procedure here was the use of specimens of different sizes so as to sample different numbers of the flaw population, thereby enabling an estimation of flaw density. The use of a notched geometry is central to this procedure and relies on a critical, but implicit, advantage of MEMS in that specimens of different geometries, *e.g*., notched, different sizes, are fabricated identically and therefore sample a single flaw population (e.g., not true of milled or sawed notches). Also critical to the overall procedure is that, mathematically, the strength distributions can be described by a single lower bound of strength, the population strength threshold, *σ*_th_. This is straightforward when the component strength distributions overlap considerably, as here, Fig. 3, or in the earlier studies of tensile specimens of slightly different lengths^13,16^, or notched specimens of slightly different geometries^16^. However, when the component strength distributions do not overlap significantly or at all, requiring extended high or low strength distribution tails, as in the earlier multi-scale bending specimen tests^33^ or comparison of notched and tensile specimen tests^16^, the analysis here is difficult to implement. In these cases, the underlying assumption that all specimens are sampling the same flaw population must be questioned.

## Conclusions

The size distributions and spatial separations of flaws in multiple layers in MEMS components were determined using a combination of dedicated strength-test specimen fabrication techniques and recent analysis linking sampled strengths to flaw population. Flaw density was verified using AFM-based topographic measurements and analysis. Critical to the experimental procedure was the use of notched bars to isolate specific flaws in addition to tensile bars to sample flaws stochastically. Critical to the analysis was the implementation of a single flaw population for both notched and tensile bars via a common fabrication process. Quantitative visualizations of the tensile bar surfaces were generated from the flaw distributions and spacings. For the MEMS structures here, the tensile strengths increased from ~2.5 GPa for the lowermost poly21 layer to 4.5 GPa for the uppermost poly4 layer. The most common flaw was spaced about 0.3 μm apart and about 35 nm in size for poly21 and about 1.5 μm apart and about 20 nm in size for poly4, although all flaw populations had extended tails such that the strength-controlling flaw sizes were about 80 nm and 40 nm, respectively, explaining the tensile strength difference. The strength and AFM measurements make clear that strength-controlling flaws in MEMS may be sparse relative to typically searched areas and expectations. Follow on work should extend these techniques to other MEMS geometries to refine the multi-specimen procedure.
